# *In silico* identification of genetic variants in glucocerebrosidase (GBA) gene involved in Gaucher's disease using multiple software tools

**DOI:** 10.3389/fgene.2014.00148

**Published:** 2014-05-27

**Authors:** Madhumathi Manickam, Palaniyandi Ravanan, Pratibha Singh, Priti Talwar

**Affiliations:** Apoptosis and Cell Death Research Laboratory, Centre for Biomedical Research, School of Biosciences and Technology, Vellore Institute of Technology UniversityVellore, India

**Keywords:** glucocerebrosidase, SIFT, MutPred, PANTHER, PMUT, PROVEAN, SNPs&GO

## Abstract

Gaucher's disease (GD) is an autosomal recessive disorder caused by the deficiency of glucocerebrosidase, a lysosomal enzyme that catalyses the hydrolysis of the glycolipid glucocerebroside to ceramide and glucose. Polymorphisms in GBA gene have been associated with the development of Gaucher disease. We hypothesize that prediction of SNPs using multiple state of the art software tools will help in increasing the confidence in identification of SNPs involved in GD. Enzyme replacement therapy is the only option for GD. Our goal is to use several state of art SNP algorithms to predict/address harmful SNPs using comparative studies. In this study seven different algorithms (SIFT, MutPred, nsSNP Analyzer, PANTHER, PMUT, PROVEAN, and SNPs&GO) were used to predict the harmful polymorphisms. Among the seven programs, SIFT found 47 nsSNPs as deleterious, MutPred found 46 nsSNPs as harmful. nsSNP Analyzer program found 43 out of 47 nsSNPs are disease causing SNPs whereas PANTHER found 32 out of 47 as highly deleterious, 22 out of 47 are classified as pathological mutations by PMUT, 44 out of 47 were predicted to be deleterious by PROVEAN server, all 47 shows the disease related mutations by SNPs&GO. Twenty two nsSNPs were commonly predicted by all the seven different algorithms. The common 22 targeted mutations are F251L, C342G, W312C, P415R, R463C, D127V, A309V, G46E, G202E, P391L, Y363C, Y205C, W378C, I402T, S366R, F397S, Y418C, P401L, G195E, W184R, R48W, and T43R.

## Introduction

Gaucher's disease (GD) is a rare genetic disease in which fatty substances accumulate in cells and certain organs (James et al., 2006). It is a common lysosomal storage disorder and results from an inborn deficiency of the enzyme glucocerebrosidase (also known as acid β-glucosidase). This enzyme is responsible for glucocerebroside (glucosylceramide) degradation. The accumulation of undegraded substrate generally happens because of enzyme deficiency, mainly within cells of the macrophage lineage or monocyte, and it is responsible for the clinical manifestations of the disease (Beutler and Grabowski, 2001). This glucosylceramide degrading enzyme is encoded by a gene named GBA, which is 7.6 kb in length and located in 1q21 locus. Recessive mutation in GBA gene affects both males and females (Horowitz et al., 1989; Zimran et al., 1991; Winfield et al., 1997). GBA protein is 497 amino acids long with the molecular weight of 55.6 KD. GBA enzyme catalyses the breakdown of glucosylceramide, a cell membrane constituent of white blood cells and red blood cells. The macrophages fail to eliminate the waste product and results in accumulation of lipids in fibrils and this turn into Gaucher cells (Aharon et al., 2004). GD can be classified into three classes namely types 1, 2, and 3. In type 1, Glycosylceramide accumulate in visceral organs whereas in type 2 and 3, the accumulation is in the central nervous system (Grabowski, 2008).

The international disease frequency of GD is 200,000 except for areas of the world with large Ashkenazi Jewish populations where 60% of the patients are estimated to be homozygous, which accounts for 75% of disease alleles (Pilar et al., 2012). Almost 300 unique mutations have been reported in the GBA gene, with distribution that spans the entire gene. These include 203 missense mutations, 18 nonsense mutations, 36 small insertions or deletions that lead to frameshift or in-frame alterations, 14 splice junction mutations and 13 complex alleles carrying two or more mutations (Hruska et al., 2008). The single nucleotide variations in the genome that occur at a frequency of more than 1% are referred as single nucleotide polymorphisms (SNPs) and in the human genome, SNPs occur in just about every 3000 base pairs (Cargill et al., 1999).

Nearly 200 mutations in the GBA gene have been described in patients with GD types 1, 2, and 3 (Jmoudiak and Futerman, 2005). L444P mutation was identified in GBA gene in patients with GD types 1, 2, and 3. The L444P substitution is one of the major SNP associated with the GBA gene. D409H, A456P, and V460V mutations were also identified in patients with GD (Tsuji et al., 1987; Latham et al., 1990). Previous findings have shown that, in 60 patients with types 1 and 3, the most common Gaucher mutations identified were N370S, L444P, and R463C. (Sidransky et al., 1994). The other mutation E326K had been identified in patients with all three types of GD, but in each instance it was found on the same allele with another GBA mutation. Also, Park et al. identified the E326K allele in 1.3% of patients with GD and in 0.9% of controls, indicating that it is a polymorphism (Park et al., 2002).

The harmful SNPs for the GBA gene have not been predicted to date *in silico*. Therefore we designed a strategy for analyzing the entire GBA coding region. Different algorithms such as SIFT (Ng and Henikoff, 2001), MutPred (Li et al., 2009), nsSNP Analyzer (Bao et al., 2005), PANTHER (Mi et al., 2012), PMUT (Costa et al., 2002), PROVEAN (Choi et al., 2012), and SNPs&GO (Calabrese et al., 2009) were utilized to predict high-risk nonsynonymous single nucleotide polymorphisms (nsSNPs) in coding regions that are likely to have an effect on the function and structure of the protein.

## Materials and methods

### Data set

SNPs associated with GBA gene were retrieved from the single nucleotide polymorphism database (dbSNP) (http://www.ncbi.nlm.nih.gov/snp/), and are commonly referred by their reference sequence IDs (rsID) (Wheeler et al., 2005).

### Validation of tolerated and deleterious SNPs

The type of genetic mutation that causes a single amino acid substitution (AAS) in a protein sequence is called nsSNP. An nsSNP could potentially influence the function of the protein, subsequently altering the phenotype of carrier. This protocol describes the use of the Sorting Intolerant From Tolerant (SIFT) algorithm (http://sift.jcvi.org) for predicting whether an AAS affects protein function. To assess the effect of a substitution, SIFT assumes that important positions in a protein sequence have been conserved throughout evolution and therefore at these positions substitutions may affect protein function. Thus, by using sequence homology, SIFT predicts the effects of all possible substitutions at each position in the protein sequence. The protocol typically takes 5–20 min, depending on the input (Kumar et al., 2009).

### Prediction of harmful mutations

MutPred (http://mutdb.org/mutpred) models structural features and functional sites changes between mutant sequences and wild-type sequence. These changes are expressed as probabilities of gain or loss of structure and function. The MutPred output contains a general score (*g*), i.e., the probability that the AAS is deleterious/disease-associated and top five property scores (*p*), where *p* is the *P*-value that certain structural and functional properties are impacted. Certain combinations of high values of general scores and low values of property scores are referred to as hypotheses (Li et al., 2009).

### Identifying disease-associated nsSNPs

nsSNP Analyzer (http://snpanalyzer.uthsc.edu) is a tool to predict whether a nsSNP has a phenotypic effect (disease-associated vs. neutral) using a machine learning method called Random Forest, and extracting structural and evolutionary information from a query nsSNP (Bao et al., 2005).

### Prediction of deleterious nsSNPs

PANTHER (http://pantherdb.org/tools/csnpScoreForm.jsp) estimates the likelihood of a particular nsSNP to cause a functional impact on a protein (Thomas et al., 2003). It calculates the substitution position-specific evolutionary conservation (subPSEC) score based on the alignment of evolutionarily related proteins. The subPSEC score is the negative logarithm of the probability ratio of the wild-type and the mutant amino acids at a particular position. The subPSEC scores are values from 0 (neutral) to about −10 (most likely to be deleterious).

### Prediction of pathological mutations on proteins

PMUT (http://mmb2.pcb.ub.es:8080/PMut) uses a robust methodology to predict disease-associated mutations. PMUT method is based on the use of neural networks (NNs) trained with a large database of neutral mutations (NEMUs) and pathological mutations of mutational hot spots, which are obtained by alanine scanning, massive mutation, and genetically accessible mutations. The final output is displayed as a pathogenicity index ranging from 0 to 1 (indexes > 0.5 single pathological mutations) and a confidence index ranging from 0 (low) to 9 (high) (Costa et al., 2005).

### Predicting the functional effect of amino acid substitutions

PROVEAN (Protein Variation Effect Analyzer) (http://provean.jcvi.org) is a sequence based predictor that estimates the effect of protein sequence variation on protein function (Choi et al., 2012). It is based on a clustering method where BLAST hits with more than 75% global sequence identity are clustered together and top 30 such clusters from a supporting sequence are averaged within and across clusters to generate the final PROVEAN score. A protein variant is predicted to be “deleterious” if the final score is below a certain threshold (default is −2.5), and is predicted to be “neutral” if the score is above the threshold.

### Prediction of disease related mutations

The SNPs&GO algorithms (http://snps-and-go.biocomp.unibo.it/snps-and-go/) predict the impact of protein variations using functional information encoded by Gene Ontology (GO) terms of the three main roots: Molecular function, Biological process, and Cellular component (Calabrese et al., 2009). SNPs&GO is a support vector machine (SVM) based web server to predict disease related mutations from the protein sequence, scoring with accuracy of 82% and Matthews correlation coefficient equal to 0.63. SNPs&GO collects, in a unique framework, information derived from protein sequence, protein sequence profile and protein functions.

## Results

### nsSNPs found by SIFT program

Protein sequence with mutational position and amino acid residue variants associated with 97 missense nsSNPs were submitted as input to the SIFT server, and the results are shown in Table 1. The lower the tolerance index, the higher the functional impact a particular amino acid residue substitution is likely to have and vice versa. Among the 97 nsSNPs analyzed, 47 nsSNPs were identified to be deleterious with a tolerance index score ≤0.05 (Kumar et al., 2009). Among 47 deleterious nsSNPs, 25 nsSNPs were found to be highly deleterious.

**Table 1:** **Tolerated and deleterious nsSNPs using SIFT**.

**S. No**	**rsID**	**Alleles**	**Position**	**AA change**	**Prediction**	**Score**
1	rs121908314	L/V	371	Leu/Val	Damaging	0.04
**2**	**rs121908313**	**F/L**	**251**	**Phe/Leu**	**Damaging**	**0.01**
3	rs121908312	K/N	79	Lys/Asn	Tolerated	0.52
4	rs121908311	G/S	377	Gly/Ser	Damaging	0.02
5	rs121908310	V/F	398	Val/Phe	Damaging	0.01
6	rs121908308	R/G	353	Arg/Gly	Tolerated	0.38
7	rs121908307	S/T	364	Ser/Thr	Tolerated	0.12
**8**	**rs121908306**	**C/G**	**342**	**Cys/Gly**	**Damaging**	**0.01**
9	rs121908305	G/R	325	Gly/Arg	Tolerated	0.44
**10**	**rs121908304**	**W/C**	**312**	**Trp/Cys**	**Damaging**	**0.00**
11	rs121908303	F/V	216	Phe/Val	Damaging	0.00
12	rs121908302	V/L	15	Val/Leu	Tolerated	0.07
13	rs121908301	G/S	478	Gly/Ser	Tolerated	0.17
14	rs121908300	Y/H	212	Tyr/His	Damaging	0.03
15	rs121908299	P/S	122	Pro/Ser	Tolerated	0.37
16	rs121908298	P/L	289	Pro/Leu	Tolerated	0.48
17	rs121908297	K/Q	157	Lys/Gln	Tolerated	0.06
**18**	**rs121908295**	**P/R**	**415**	**Pro/Arg**	**Damaging**	**0.00**
19	rs80356773	R/H	496	Arg/His	Tolerated	0.19
20	rs80356772	R/H	463	Arg/His	Tolerated	0.06
**21**	**rs80356771**	**R/C**	**463**	**Arg/Cys**	**Damaging**	**0.02**
22	rs80356769	V/L	394	Val/Leu	Damaging	0.03
23	rs80356765	A/T	338	Ala/Thr	Tolerated	0.39
24	rs80356763	R/L	131	Arg/Leu	Tolerated	0.24
25	rs80205046	P/L	182	Pro/Leu	Damaging	0.00
26	rs80116658	G/D	265	Gly/Asp	Damaging	0.00
27	rs80020805	M/I	416	Met/Ile	Tolerated	0.42
28	rs79945741	F/L	213	Phe/Leu	Tolerated	0.18
**29**	**rs79796061**	**D/V**	**127**	**Asp/Val**	**Damaging**	**0.00**
30	rs79696831	R/H	285	Arg/His	Damaging	0.00
31	rs79653797	R/Q	120	Arg/Gln	Damaging	0.00
32	rs79637617	P/L	122	Pro/Leu	Damaging	0.02
33	rs79215220	P/R	266	Pro/Arg	Damaging	0.00
34	rs79185870	F/L	417	Phe/Leu	Damaging	0.01
35	rs78973108	R/Q	257	Arg/Gln	Tolerated	0.05
36	rs78911246	G/V	189	Gly/Val	Damaging	0.02
37	rs78802049	D/E	409	Asp/Glu	Tolerated	0.32
38	rs78769774	R/Q	48	Arg/Gln	Tolerated	0.06
39	rs78715199	D/E	380	Asp/Glu	Damaging	0.00
**40**	**rs78396650**	**A/V**	**309**	**Ala/Val**	**Damaging**	**0.00**
41	rs78198234	H/R	311	His/Arg	Damaging	0.00
42	rs78188205	A/D	318	Ala/Asp	Tolerated	0.63
43	rs77959976	M/I	123	Met/Ile	Tolerated	1.00
44	rs77834747	I/S	119	Ile/Ser	Tolerated	0.34
**45**	**rs77829017**	**G/E**	**46**	**Gly/Glu**	**Damaging**	**0.01**
46	rs77738682	N/I	392	Asn/Ile	Damaging	0.00
**47**	**rs77451368**	**G/E**	**202**	**Gly/Glu**	**Damaging**	**0.02**
48	rs77369218	D/V	409	Asp/Val	Tolerated	0.06
49	rs77321207	Y/C	395	Tyr/Cys	Damaging	0.00
50	rs77284004	D/A	380	Asp/Ala	Damaging	0.00
51	rs77035024	F/L	411	Phe/Leu	Tolerated	0.30
52	rs77019233	N/K	117	Asn/Lys	Tolerated	0.21
**53**	**rs76910485**	**P/L**	**391**	**Pro/Leu**	**Damaging**	**0.00**
54	rs76763715	N/S	370	Asn/Ser	Damaging	0.05
55	rs76763715	N/T	370	Asn/Thr	Damaging	0.04
56	rs76539814	T/I	323	Thr/Ile	Tolerated	0.48
**57**	**rs76228122**	**Y/C**	**363**	**Tyr/Cys**	**Damaging**	**0.00**
**58**	**rs76026102**	**Y/C**	**205**	**Tyr/Cys**	**Damaging**	**0.00**
**59**	**rs76014919**	**W/C**	**378**	**Trp/Cys**	**Damaging**	**0.00**
60	rs75954905	F/L	37	Phe/Leu	Tolerated	0.30
61	rs75671029	D/N	443	Asp/Asn	Tolerated	0.93
62	rs75636769	A/E	190	Ala/Glu	Tolerated	1.00
**63**	**rs75564605**	**I/T**	**402**	**Ile/Thr**	**Damaging**	**0.04**
64	rs75548401	T/M	369	Thr/Met	Tolerated	0.08
**65**	**rs75528494**	**S/R**	**366**	**Ser/Arg**	**Damaging**	**0.03**
66	rs75385858	N/T	396	Asn/Thr	Damaging	0.00
**67**	**rs75243000**	**F/S**	**397**	**Phe/Ser**	**Damaging**	**0.02**
68	rs75090908	D/E	399	Asp/Glu	Tolerated	0.17
69	rs74979486	R/Q	359	Arg/Gln	Tolerated	0.05
70	rs74953658	D/E	24	Asp/Glu	Damaging	0.01
**71**	**rs74752878**	**Y/C**	**418**	**Tyr/Cys**	**Damaging**	**0.00**
72	rs74731340	S/N	271	Ser/Asn	Tolerated	0.26
**73**	**rs74598136**	**P/L**	**401**	**Pro/Leu**	**Damaging**	**0.00**
74	rs74500255	F/Y	216	Phe/Tyr	Tolerated	0.34
**75**	**rs74462743**	**G/E**	**195**	**Gly/Glu**	**Damaging**	**0.00**
**76**	**rs61748906**	**W/R**	**184**	**Trp/Arg**	**Damaging**	**0.00**
77	rs11558184	R/Q	353	Arg/Gln	Tolerated	0.59
78	rs2230288	E/K	326	Glu/Lys	Tolerated	0.86
79	rs1141820	H/R	60	His/Arg	Tolerated	0.54
80	rs1141818	H/Y	60	His/Tyr	Tolerated	0.09
81	rs1141815	M/T	53	Met/Thr	Tolerated	0.59
**82**	**rs1141814**	**R/W**	**48**	**Arg/Trp**	**Damaging**	**0.00**
83	rs1141812	R/S	44	Arg/Ser	Tolerated	0.14
84	rs1141811	T/I	43	Thr/Ile	Damaging	0.01
**85**	**rs1141811**	**T/R**	**43**	**Thr/Arg**	**Damaging**	**0.02**
86	rs1141808	E/K	41	Glu/Lys	Tolerated	0.52
87	rs1141804	S/G	16	Ser/Gly	Tolerated	1.00
88	rs1141802	L/S	15	Leu/Ser	Tolerated	0.63
89	rs1064651	D/H	409	Asp/His	Tolerated	0.05
90	rs1064648	R/H	329	Arg/His	Tolerated	0.17
91	rs1064644	S/P	196	Ser/Pro	Tolerated	0.17
92	rs421016	L/P	444	Leu/Pro	Damaging	0.00
93	rs381737	F/I	213	Phe/Ile	Tolerated	0.18
94	rs381427	V/E	191	Val/Glu	Tolerated	0.16
95	rs381427	V/G	191	Val/Gly	Tolerated	0.16
96	rs368060	A/P	456	Ala/Pro	Tolerated	0.09
97	rs364897	N/S	188	Asn/Ser	Tolerated	0.17

The consensus SNPs are shown in bold.

### Validation of harmful mutations

The MutPred score is the probability that an AAS is deleterious/disease-associated. A missense mutation with a MutPred score >0.5 could be considered as “harmful,” while a MutPred score >0.75 should be considered a high confidence “harmful” prediction (Li et al., 2009). Among the 47 deleterious nsSNPs, 8 were found to be harmful mutations with a score of >0.5 and <0.75 and 38 were found to be high confidence (highly harmful) mutations and 1 nsSNP found to be normal with the score of 0.193 (Table 2).

**Table 2:** **Prediction of functional effects of nsSNPs using MutPred**.

**S. No**	**rsID**	**Alleles**	**Position**	**AA change**	**MutPred prediction**	**Score**
1	rs121908314	L/V	371	Leu/val	High confidence	0.824
**2**	**rs121908313**	**F/L**	**251**	**Phe/Leu**	**High confidence**	**0.778**
3	rs121908311	G/S	377	Gly/Ser	Neutral	0.193
4	rs121908310	V/F	298	Val/Phe	High confidence	0.765
**5**	**rs121908306**	**C/G**	**342**	**Cys/Gly**	**High confidence**	**0.792**
**6**	**rs121908304**	**W/C**	**312**	**Trp/Cys**	**Harmful mutation**	**0.735**
7	rs121908303	F/V	216	Phe/Val	High confidence	0.879
8	rs121908300	Y/H	212	Tyr/His	High confidence	0.82
**9**	**rs121908295**	**P/R**	**415**	**Pro/Arg**	**High confidence**	**0.914**
**10**	**rs80356771**	**R/C**	**463**	**Arg/Cys**	**Harmful mutation**	**0.664**
11	rs80356769	V/L	394	Val/Leu	High confidence	0.794
12	rs80205046	P/L	182	Pro/Leu	High confidence	0.892
13	rs80116658	G/D	265	Gly/Asp	High confidence	0.963
**14**	**rs79796061**	**D/V**	**127**	**Asp/Val**	**High confidence**	**0.754**
15	rs79696831	R/H	285	Arg/His	High confidence	0.884
16	rs79653797	R/Q	120	Arg/Gln	High confidence	0.902
17	rs79637617	P/L	122	Pro/Leu	High confidence	0.835
18	rs79215220	P/R	166	Pro/Arg	High confidence	0.836
19	rs79185870	F/L	417	Phe/Leu	High confidence	0.905
20	rs78911246	G/V	189	Gly/Val	Harmful mutation	0.713
21	rs78715199	D/E	380	Asp/Glu	High confidence	0.837
**22**	**rs78396650**	**A/V**	**309**	**Ala/Val**	**High confidence**	**0.776**
23	rs78198234	H/R	311	His/Arg	High confidence	0.873
**24**	**rs77829017**	**G/E**	**46**	**Gly/Glu**	**High confidence**	**0.856**
25	rs77738682	N/I	392	Asn/Ile	High confidence	0.814
**26**	**rs77451368**	**G/E**	**202**	**Gly/Glu**	**Harmful mutation**	**0.676**
27	rs77321207	Y/C	304	Tyr/Cys	High confidence	0.909
28	rs77284004	D/A	380	Asp/Ala	High confidence	0.872
**29**	**rs76910485**	**P/L**	**391**	**Pro/Leu**	**High confidence**	**0.889**
30	rs76763715	N/S	370	Ans/Ser	High confidence	0.876
31	rs76763715	N/T	370	Asn/Thr	High confidence	0.89
**32**	**rs76228122**	**Y/C**	**363**	**Tyr/Cys**	**High confidence**	**0.93**
**33**	**rs76026102**	**Y/C**	**205**	**Tyr/Cys**	**High confidence**	**0.857**
**34**	**rs76014919**	**W/C**	**378**	**Trp/Cys**	**High confidence**	**0.842**
**35**	**rs75564605**	**I/T**	**402**	**IleThr**	**High confidence**	**0.838**
**36**	**rs75528494**	**S/R**	**366**	**Ser/Arg**	**Harmful mutation**	**0.681**
37	rs75385858	N/T	396	Asn/Thr	High confidence	0.848
**38**	**rs75243000**	**F/S**	**397**	**Phe/Ser**	**Harmful mutation**	**0.724**
39	rs74953658	D/E	24	Asp/Glu	High confidence	0.818
**40**	**rs74752878**	**Y/C**	**418**	**Tyr/Cys**	**High confidence**	**0.872**
**41**	**rs74598136**	**P/L**	**401**	**Pro/Leu**	**High confidence**	**0.888**
**42**	**rs74462743**	**G/E**	**195**	**Gly/Glu**	**High confidence**	**0.859**
**43**	**rs61748906**	**W/R**	**184**	**Trp/Arg**	**High confidence**	**0.902**
**44**	**rs1141814**	**R/W**	**48**	**Arg/Trp**	**High confidence**	**0.804**
45	rs1141811	T/I	43	Thr/Ile	Harmful mutation	0.504
**46**	**rs1141811**	**T/R**	**43**	**Thr/Arg**	**Harmful mutation**	**0.579**
47	rs421016	L/P	444	Leu/Pro	High confidence	0.899

The consensus SNPs are shown in bold.

### Disease-associated nsSNPs

Out of 47 deleterious nsSNPs, 43 were found to be a disease causing nsSNPs and 4 were found to be neutral nsSNPs (Table 3).

**Table 3:** **The results from nsSNP Analyzer, PMUT, PROVEAN, and SNPs&GO**.

**S. No**	**rsID**	**Allele**	**Position**	**AA change**	**nsSNP Analyzer**	**PMUT**	**PROVEAN**		**SNPs&GO**
							**Score**	**Prediction**	
1	rs121908314	L/V	371	Leu/val	Neutral	Neutral	−2.331	Neutral	Disease
**2**	**rs121908313**	**F/L**	**251**	**Phe/Leu**	**Disease**	**Pathological**	**−4.567**	**Deleterious**	**Disease**
3	rs121908311	G/S	377	Gly/Ser	Disease	Neutral	−5.128	Deleterious	Disease
4	rs121908310	V/F	398	Val/Phe	Disease	Neutral	−4.185	Deleterious	Disease
**5**	**rs121908306**	**C/G**	**342**	**Cys/Gly**	**Disease**	**Pathological**	**−11.467**	**Deleterious**	**Disease**
**6**	**rs121908304**	**W/C**	**312**	**Trp/Cys**	**Disease**	**Pathological**	**−12.258**	**Deleterious**	**Disease**
7	rs121908303	F/V	216	Phe/Val	Disease	Neutral	−7	Deleterious	Disease
8	rs121908300	Y/H	212	Tyr/His	Disease	Neutral	−4.267	Deleterious	Disease
**9**	**rs121908295**	**P/R**	**415**	**Pro/Arg**	**Disease**	**Pathological**	**−8.793**	**Deleterious**	**Disease**
**10**	**rs80356771**	**R/C**	**463**	**Arg/Cys**	**Disease**	**Pathological**	**−5.279**	**Deleterious**	**Disease**
11	rs80356769	V/L	394	Val/Leu	Neutral	Neutral	−2.031	Neutral	Disease
12	rs80205046	P/L	182	Pro/Leu	Disease	Neutral	−9.917	Deleterious	Disease
13	rs80116658	G/D	265	Gly/Asp	Disease	Neutral	−6.442	Deleterious	Disease
**14**	**rs79796061**	**D/V**	**127**	**Asp/Val**	**Disease**	**Pathological**	**−8.625**	**Deleterious**	**Disease**
15	rs79696831	R/H	285	Arg/His	Disease	Neutral	−4.792	Deleterious	Disease
16	rs79653797	R/Q	120	Arg/Gln	Disease	Neutral	−3.641	Deleterious	Disease
17	rs79637617	P/L	122	Pro/Leu	Disease	Neutral	−9.265	Deleterious	Disease
18	rs79215220	P/R	266	Pro/Arg	Disease	Neutral	−8.275	Deleterious	Disease
19	rs79185870	F/L	417	Phe/Leu	Disease	Neutral	−5.095	Deleterious	Disease
20	rs78911246	G/V	189	Gly/Val	Disease	Neutral	−6.4	Deleterious	Disease
21	rs78715199	D/E	380	Asp/Glu	Neutral	Neutral	−3.797	Deleterious	Disease
**22**	**rs78396650**	**A/V**	**309**	**Ala/Val**	**Disease**	**Pathological**	**−3.533**	**Deleterious**	**Disease**
23	rs78198234	H/R	311	His/Arg	Disease	Neutral	−7.667	Deleterious	Disease
**24**	**rs77829017**	**G/E**	**46**	**Gly/Glu**	**Disease**	**Pathological**	**−5.925**	**Deleterious**	**Disease**
25	rs77738682	N/I	392	Asn/Ile	Disease	Neutral	−7.593	Deleterious	Disease
**26**	**rs77451368**	**G/E**	**202**	**Gly/Glu**	**Disease**	**Pathological**	**−5.178**	**Deleterious**	**Disease**
27	rs77321207	Y/C	304	Tyr/Cys	Disease	Neutral	−8.358	Deleterious	Disease
28	rs77284004	D/A	380	Asp/Ala	Disease	Neutral	−7.593	Deleterious	Disease
**29**	**rs76910485**	**P/L**	**391**	**Pro/Leu**	**Disease**	**Pathological**	**−9.269**	**Deleterious**	**Disease**
30	rs76763715	N/S	370	Ans/Ser	Neutral	Neutral	−2.128	Neutral	Disease
31	rs76763715	N/T	370	Asn/Thr	Disease	Neutral	−3.062	Deleterious	Disease
**32**	**rs76228122**	**Y/C**	**363**	**Tyr/Cys**	**Disease**	**Pathological**	**−8.492**	**Deleterious**	**Disease**
**33**	**rs76026102**	**Y/C**	**205**	**Tyr/Cys**	**Disease**	**Pathological**	**−7.552**	**Deleterious**	**Disease**
**34**	**rs76014919**	**W/C**	**378**	**Trp/Cys**	**Disease**	**Pathological**	**−12.306**	**Deleterious**	**Disease**
**35**	**rs75564605**	**I/T**	**402**	**IleThr**	**Disease**	**Pathological**	**−4.363**	**Deleterious**	**Disease**
**36**	**rs75528494**	**S/R**	**366**	**Ser/Arg**	**Disease**	**Pathological**	**−2.806**	**Deleterious**	**Disease**
37	rs75385858	N/T	396	Asn/Thr	Disease	Neutral	−5.562	Deleterious	Disease
**38**	**rs75243000**	**F/S**	**397**	**Phe/Ser**	**Disease**	**Pathological**	**−4.782**	**Deleterious**	**Disease**
39	rs74953658	D/E	24	Asp/Glu	Disease	Neutral	−3.037	Deleterious	Disease
**40**	**rs74752878**	**Y/C**	**418**	**Tyr/Cys**	**Disease**	**Pathological**	**−8.526**	**Deleterious**	**Disease**
**41**	**rs74598136**	**P/L**	**401**	**Pro/Leu**	**Disease**	**Pathological**	**−8.136**	**Deleterious**	**Disease**
**42**	**rs74462743**	**G/E**	**195**	**Gly/Glu**	**Disease**	**Pathological**	**−7.767**	**Deleterious**	**Disease**
**43**	**rs61748906**	**W/R**	**184**	**Trp/Arg**	**Disease**	**Pathological**	**−13.028**	**Deleterious**	**Disease**
**44**	**rs1141814**	**R/W**	**48**	**Arg/Trp**	**Disease**	**Pathological**	**−-6.879**	**Deleterious**	**Disease**
45	rs1141811	T/I	43	Thr/Ile	Disease	Neutral	−3.515	Deleterious	Disease
**46**	**rs1141811**	**T/R**	**43**	**Thr/Arg**	**Disease**	**Pathological**	**−2.557**	**Deleterious**	**Disease**
47	rs421016	L/P	444	Leu/Pro	Disease	Neutral	−4.995	Deleterious	Disease

The consensus SNPs are shown in bold.

### Validation by panther

The protein sequence was given as input and analyzed for the deleterious effect on protein function. The subPSEC scores are values from 0 (neutral) to about −10 (deleterious) (Thomas et al., 2003). Out of 47 deleterious nsSNPs, 8 were found to be more than −6 (highly deleterious) and rest were found to be less deleterious. The mutant with a greater *P*_deleterious_ tends to have more severe destructions in function. It was found that 32 out of 47 deleterious nsSNPs scored greater than 3 and rests were below the damage threshold (Table 4).

**Table 4:** **Mutant scores from PANTHER**.

**S. NO**	**rsID**	**Alleles**	**Position**	**AA change**	**subPSEC**	**P_deleterious_**
1	rs121908314	L/V	371	Leu/val	−3.34802	0.58614
**2**	**rs121908313**	**F/L**	**251**	**Phe/Leu**	**−2.59088**	**0.39912**
3	rs121908311	G/S	377	Gly/Ser	−5.35062	0.91298
4	rs121908310	V/F	398	Val/Phe	−3.36629	0.59056
**5**	**rs121908306**	**C/G**	**342**	**Cys/Gly**	**−3.57193**	**0.63921**
**6**	**rs121908304**	**W/C**	**312**	**Trp/Cys**	**−2.59838**	**0.40092**
7	rs121908303	F/V	216	Phe/Val	−4.88341	0.868
8	rs121908300	Y/H	212	Tyr/His	−5.32716	0.9111
**9**	**rs121908295**	**P/R**	**415**	**Pro/Arg**	**−4.90228**	**0.87015**
**10**	**rs80356771**	**R/C**	**463**	**Arg/Cys**	**−4.45218**	**0.81033**
11	rs80356769	V/L	394	Val/Leu	−2.8436	0.46098
12	rs80205046	P/L	182	Pro/Leu	−6.3153	0.96495
13	rs80116658	G/D	265	Gly/Asp	−6.00914	0.95299
**14**	**rs79796061**	**D/V**	**127**	**Asp/Val**	**−6.29967**	**0.96442**
15	rs79696831	R/H	285	Arg/His	−4.32962	0.79078
16	rs79653797	R/Q	120	Arg/Gln	−4.52062	0.82063
17	rs79637617	P/L	122	Pro/Leu	−4.49053	0.81616
18	rs79215220	P/R	266	Pro/Arg	−6.27743	0.96365
19	rs79185870	F/L	417	Phe/Leu	−4.16977	0.7631
20	rs78911246	G/V	189	Gly/Val	−3.38537	0.59517
21	rs78715199	D/E	380	Asp/Glu	−2.02623	0.27413
**22**	**rs78396650**	**A/V**	**309**	**Ala/Val**	**−4.2769**	**0.78192**
23	rs78198234	H/R	311	His/Arg	−4.57198	0.82807
**24**	**rs77829017**	**G/E**	**46**	**Gly/Glu**	**−5.04065**	**0.885**
25	rs77738682	N/I	392	Asn/Ile	−4.02188	0.73534
**26**	**rs77451368**	**G/E**	**202**	**Gly/Glu**	**−1.32995**	**0.15842**
27	rs77321207	Y/C	304	Tyr/Cys	−6.26737	0.96329
28	rs77284004	D/A	380	Asp/Ala	−2.36947	0.34739
**29**	**rs76910485**	**P/L**	**391**	**Pro/Leu**	**−6.12534**	**0.95793**
30	rs76763715	N/S	370	Ans/Ser	−2.69603	0.42459
31	rs76763715	N/T	370	Asn/Thr	−1.97735	0.26451
**32**	**rs76228122**	**Y/C**	**363**	**Tyr/Cys**	**−4.75749**	**0.85289**
**33**	**rs76026102**	**Y/C**	**205**	**Tyr/Cys**	**−5.89294**	**0.9475**
**34**	**rs76014919**	**W/C**	**378**	**Trp/Cys**	**−5.31772**	**0.91033**
**35**	**rs75564605**	**I/T**	**402**	**IleThr**	**−3.78009**	**0.6857**
**36**	**rs75528494**	**S/R**	**366**	**Ser/Arg**	**−2.07688**	**0.28432**
37	rs75385858	N/T	396	Asn/Thr	−3.61569	0.64924
**38**	**rs75243000**	**F/S**	**397**	**Phe/Ser**	**−2.88329**	**0.47086**
39	rs74953658	D/E	24	Asp/Glu	−4.17446	0.76395
**40**	**rs74752878**	**Y/C**	**418**	**Tyr/Cys**	**−6.31864**	**0.96506**
**41**	**rs74598136**	**P/L**	**401**	**Pro/Leu**	**−2.14888**	**0.2992**
**42**	**rs74462743**	**G/E**	**195**	**Gly/Glu**	**−4.74669**	**0.85153**
**43**	**rs61748906**	**W/R**	**184**	**Trp/Arg**	**−3.5793**	**0.64091**
**44**	**rs1141814**	**R/W**	**48**	**Arg/Trp**	**−7.03366**	**0.9826**
45	rs1141811	T/I	43	Thr/Ile	−4.20869	0.77007
**46**	**rs1141811**	**T/R**	**43**	**Thr/Arg**	**−4.05221**	**0.7412**
47	rs421016	L/P	444	Leu/Pro	−3.43747	0.60766

The consensus SNPs are shown in bold.

### Functional impact of mutations on proteins

The functional impact of 47 deleterious nsSNPs in protein of GBA was analyzed using PMUT server. Of the 47 nsSNPs, 22 are classified as pathological, and the remaining were neutral (Table 3).

### Protein variation effect analysis

PROVEAN predicts the effect of the variant on the biological function of the protein based on sequence homology. PROVEAN scores are classified as “deleterious” if below a certain threshold (here −2.5) and “neutral” if above it (Choi et al., 2012). Out of 47 nsSNPs, 44 were predicted to be “deleterious” and 3 were found to be “neutral” (Table 3).

### Prediction of disease related mutations by SNPs&GO

SNPs&GO is trained and tested with cross-validation procedures in which similar proteins are placed together as a dataset to calculate the *LGO* score derived from the GO data base. All 47 deleterious nsSNPs showed the disease related mutations (Table 3).

## Discussion

**Figure 1:**
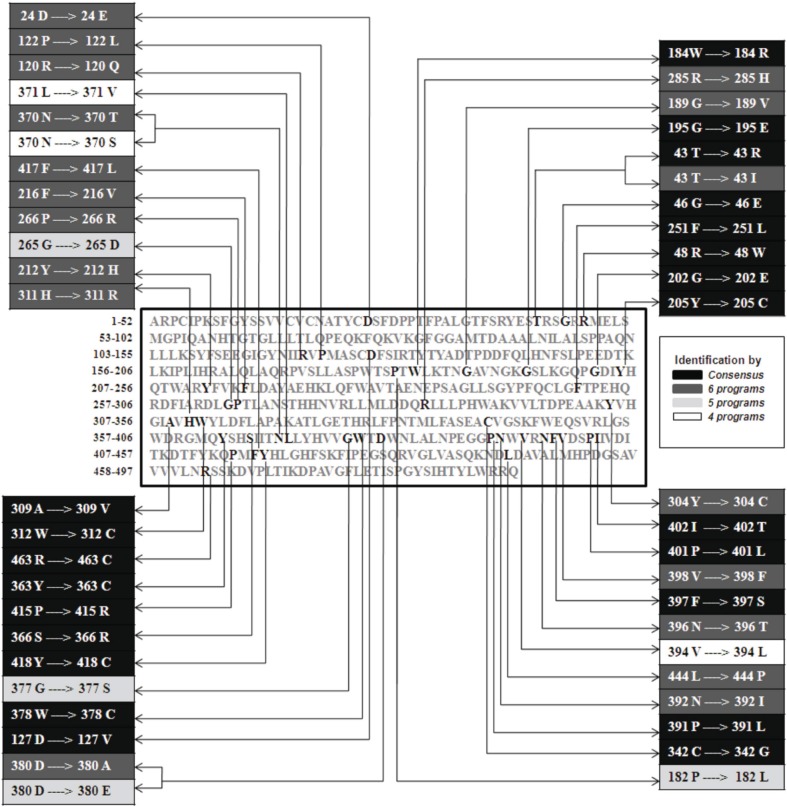
**Sets of various mutations identified using various software tools**. The respective locations of 44 amino acids responsible for all 47 mutations are shown in the sequence (center, colored in bold) and 22 common mutations are highlighted as consensus.

In the recent years, SNPs have emerged as the new generation molecular markers. The harmful SNPs for the GBA gene were never been predicted to date *in silico*. This study was designed to understand the genetic variations associated with GBA gene. We have predicted the harmful nsSNPs using SIFT, MutPred, nsSNP Analyzer, PANTHER, PMUT, PROVEAN, and SNPs&GO state of the art computational tools. Among 97 nsSNPs, 47 were found to be deleterious with a tolerance index score of ≤0.05 found by SIFT program. Among the 47 deleterious nsSNPs, 46 were found to be harmful nsSNPs found by MutPred, 43 were found to be disease causing nsSNPs by nsSNP Analyzer tool, 32 are highly deleterious found by PANTHER program, 22 are classified as pathological mutations by PMUT, 44 were predicted to be deleterious by PROVEAN server while all 47 deleterious nsSNPs showed the disease related mutations by SNPs&GO. Also, we found that SNPs&GO was most successful of all state of the art SNP prediction programs that were used for this comparative study. In this work, we found 22 nsSNPs that are common in all (SIFT, MutPred, nsSNP Analyzer, PANTHER, PMUT, PROVEAN, and SNPs&GO) prediction (Figure 1). These sets of 22 nsSNPs (F251L, C342G, W312C, P415R, R463C, D127V, A309V, G46E, G202E, P391L, Y363C, Y205C, W378C, I402T, S366R, F397S, Y418C, P401L, G195E, W184R, R48W, and T43R) are possibly the main targeted mutation for the GD (Tables 1–4). The previous work has shown that, in 60 patients with types 1 and 3, the most common Gaucher mutations identified were L444P, N370S, and R463C. L444P was the most common mutation in GD types 1, 2, and 3 (Latham et al., 1990; Sidransky et al., 1994). In our analysis, out of 7 methods, 6 methods (Sift, MutPred, PROVEAN, PANTHER, nsSNP Analyzer, and SNPs&GO) shows L444P mutation as damaging, 3 methods shows N370S mutation as damaging and all the 7 methods shows R463C mutation as damaging. D409H, A456P, E326K, and V460V mutations were also identified in patients with GD (Tsuji et al., 1987; Park et al., 2002). In our analysis SIFT result shows D409H, A456P, and E326K mutation is the tolerated mutation. Further studies using these mutations will shed light on the genetic understanding of this major lysosomal storage disease.

## Author contributions

Madhumathi Manickam, Priti Talwar, and Palaniyandi Ravanan wrote the main manuscript and analyzed original datasets. Pratibha Singh prepared tables and figure. All authors reviewed the manuscript.

### Conflict of interest statement

The authors declare that the research was conducted in the absence of any commercial or financial relationships that could be construed as a potential conflict of interest.
